# Prevalence, Incidence, and Sensitization Profile of β-lactam Antibiotic Allergy in Hong Kong

**DOI:** 10.1001/jamanetworkopen.2020.4199

**Published:** 2020-05-06

**Authors:** Philip H. Li, Heather H. F. Yeung, Chak-Sing Lau, Elaine Y. L. Au

**Affiliations:** 1 Division of Rheumatology and Clinical Immunology, Department of Medicine, Queen Mary Hospital, Hong Kong; 2 Division of Clinical Immunology, Department of Pathology, Queen Mary Hospital, Hong Kong

## Abstract

**Importance:**

Allergy to β-lactam antibiotics is one of the most frequently reported drug reactions, but epidemiological data in Chinese populations are lacking. Ethnic- and region-specific sensitization patterns of skin testing for β-lactam antibiotic allergy are also unknown.

**Objective:**

To identify the prevalence, 1-year incidence, and sensitization patterns of β-lactam antibiotic allergy in patients in Hong Kong.

**Design, Setting, and Participants:**

This cross-sectional study obtained territorywide, anonymized electronic patient data from the Clinical Management Systems of the Hospital Authority, the sole publicly funded health care system in Hong Kong with facilities in 7 regions (Hong Kong East, Hong Kong West, Kowloon Central, Kowloon East, Kowloon West, New Territories East, and New Territories West). All referrals to Queen Mary Hospital for β-lactam antibiotic allergy testing from January 1, 2018, to December 31, 2019, were also analyzed for sensitization patterns.

**Main Outcomes and Measures:**

Prevalence and cumulative incidence of β-lactam antibiotic allergy reported in Hong Kong, and sensitization patterns according to β-lactam antibiotic allergy skin testing.

**Results:**

Complete records of 7 184 271 unique patients were analyzed, with a men to women ratio of 1:1.2 and with a median age of 44 years. The prevalence of physician-reported β-lactam antibiotic allergy was 2.0%, and the cumulative incidence was 107 per 100 000 population. Of the 34 402 new drug allergies reported in 2018, 8032 (23.3%) were β-lactam antibiotic allergies. Three hundred fifty-five patients with reactions suggestive of β-lactam antibiotic allergy underwent skin testing, and only 49 (13.8%; 95% CI, 10.64%-17.90%) of them had positive test results. Of these 49 patients, 14 (28.6%; 95% CI, 18.35%-44.49%) had selective reaction and 35 (71.4%; 95% CI, 59.84%-85.27%) had nonselective reaction. The sensitization rate to either benzylpenicilloyl polylysine or a minor determinant (benzylpenicilloate) was 47.0% (n = 23; 95% CI, 34.85%-63.21%), with 10 patients monosensitized to benzylpenicilloyl polylysine only (20.4%; 95% CI, 11.74%-35.48%) and 5 to benzylpenicilloate only (10.2%; 95% CI, 4.45%-23.42%).

**Conclusions and Relevance:**

Results of this study suggest that patients in Hong Kong with β-lactam antibiotic allergy had much higher rates of monosensitization to benzylpenicilloyl polylysine and benzylpenicilloate, making these reagents essential in β-lactam antibiotic skin tests. Such a finding warrants future studies into whether this sensitization is specific to ethnicity or region.

## Key Points

**Question:**

What are the prevalence, incidence, and sensitization patterns of β-lactam antibiotic allergy in Hong Kong?

**Findings:**

In this cross-sectional study of 7 184 271 patient records, the prevalence of reported β-lactam antibiotic allergy was 2.0% and the cumulative incidence was 107 per 100 000 population. Only 13.8% of patients who underwent skin testing had positive results, but 47% had high rates of sensitization to the reagent benzylpenicilloyl polylysine or a minor determinant (benzylpenicilloate).

**Meaning:**

These findings suggest that benzylpenicilloyl polylysine and a minor determinant (benzylpenicilloate) should be included in β-lactam antibiotic allergy skin testing for the large and rapidly growing burden of β-lactam antibiotic allergies reported among patients in Hong Kong.

## Teaser

This cross-sectional study examines the reported penicillin allergies and sensitization to skin-testing compounds benzylpenicilloyl polylysine and minor determinants among patients in Hong Kong.

## Introduction

β-lactam antibiotics, including penicillin, cephalosporin, carbapenem, and monobactam, are the most widely used class of antibiotics and most frequently associated with drug allergy.^1^ The prevalence of penicillin allergy in western populations has been estimated at approximately 10%.^2,3,4,5^ However, many patients mistakenly self-report non–immune-mediated adverse drug reactions as allergy, and up to 90% of these patients are found not to be genuinely allergic after evaluation.^6,7,8,9,10^ Misreported β-lactam antibiotic allergies are associated with obligatory use of less effective antibiotics and a multitude of adverse clinical consequences.^11,12,13,14^ Epidemiological data are crucial for guiding urgently needed β-lactam antibiotic allergy testing, especially against the growing pandemic of antimicrobial resistance.^15^

Evaluation of reactions suggestive of β-lactam antibiotic allergy includes history taking, skin tests (including skin prick and intradermal), and drug provocation tests.^16,17^ Skin tests in Europe and Hong Kong are performed using a commercially available kit with benzylpenicilloyl polylysine (PPL) and the minor determinant (MD) benzylpenicilloate (diagnostic allergy penicillin; Diater), in addition to benzylpenicillin, amoxicillin, and the index culprit β-lactam antibiotics (if available). However, the use of the major and minor antigenic determinants in modern β-lactam antibiotic allergy skin tests remains controversial. For example, some studies have shown comparable negative predictive values without MD and/or PPL, whereas other studies have reported that omission of MD could miss up to 20% of patients with penicillin allergy.^18,19^ Marked variations in patterns of sensitization are also likely among different populations, with fewer patients monosensitized to PPL in Europe and a decline in positive skin test results in the United States.^20,21,22^ Ethnic- and region-specific data on sensitization patterns are needed to determine optimal local β-lactam antibiotic skin test strategies, but data from Chinese cohorts are lacking.

A pilot study conducted in Hong Kong found a 5% prevalence of misreported β-lactam antibiotic allergy in a cohort of hospitalized patients and estimated a 0.5% prevalence of genuine allergy.^8^ However, that pilot study was small and based on admissions to a single hospital over a 6-month period.^8^ The overall and absolute prevalence of misreported β-lactam antibiotic allergy remains unknown. Furthermore, few studies have looked into the incidence rather than just prevalence of reported β-lactam antibiotic allergy; that is, how many new reports of β-lactam antibiotic allergy are being generated over time. Such data would be of immense advantage in further delineating the rate of the rapidly increasing need for β-lactam antibiotic allergy testing.

To elucidate these areas of uncertainty, we used the electronic records system of the Hospital Authority in Hong Kong, which has one of the world’s largest clinical information systems with a unified drug allergy record database for more than 7.1 million unique patients across the entire territory, to examine the near-absolute prevalence and annual incidence of β-lactam antibiotic allergy in Hong Kong. We also investigated the sensitization patterns to identify the sensitization rate of the major and minor antigenic determinants in skin tests for patients with β-lactam antibiotic allergy.

## Methods

In this cross-sectional study, we retrieved anonymized data from the Hospital Authority Clinical Management Systems in Hong Kong. Skin test results for all available medical records of patients referred to Queen Mary Hospital for β-lactam antibiotic allergy testing were also extracted. Informed consent was waived (because all data were anonymous and collected retrospectively) and data extraction was approved by the institutional review board of the University of Hong Kong and Hospital Authority Hong Kong West cluster. This report followed the Strengthening the Reporting of Observational Studies in Epidemiology (STROBE) reporting guideline.^23^

The Hospital Authority is the sole publicly funded health care system in Hong Kong that serves a population of more than 7 million patients through 43 hospitals, 49 specialist outpatient clinics, and 73 general outpatient clinics. These facilities are organized into 7 clusters (Hong Kong East, Hong Kong West, Kowloon Central, Kowloon East, Kowloon West, New Territories East, and New Territories West) on the basis of geographical locations and provide approximately 90% of inpatient care in Hong Kong.^24,25^ Data for the present study were retrieved and analyzed with assistance from the Information Technology and Health Informatics Division at the Hospital Authority Head Office. All available records with complete allergy data were eligible and analyzed for report of any drug allergy or any allergy to β-lactam antibiotics (ie, penicillin, cephalosporin, carbapenem, or monobactam). Only patients whose attending physicians entered allergy data (ie, confirming no known drug allergy, or completing drug allergy record) were included in the analysis. Cross-sectional data from all available patient records were retrieved on September 15, 2019; the point prevalence of β-lactam antibiotic allergy reports in Hong Kong was based on the data on that day. Data from January 1, 2018, to December 31, 2018, were also retrieved for calculating the incidence of drug allergy and β-lactam antibiotic allergy within the year.

To study the sensitization pattern of patients with β-lactam antibiotic allergy in the same period, we analyzed the skin test results for all available medical records of patients referred to Queen Mary Hospital for β-lactam antibiotic allergy testing between January 1, 2018, and December 31, 2019. All patients who were referred and gave consent for β-lactam antibiotic allergy testing, regardless of any foreseeable need for β-lactam antibiotics in the future, were offered skin prick and intradermal tests. All patients who underwent testing were also under the care of the Hospital Authority and therefore were part of this cross-sectional study. Skin tests were performed in accordance to the British Society for Allergy and Clinical Immunology standards and the Hong Kong Institute of Allergy guidelines.^17,26^ Since mid-2018, Queen Mary Hospital has been the only referral center with formal immunology or allergy testing services under the Hospital Authority. Queen Mary Hospital receives allergy referrals from across the entire territory, and its patients represented the general population of Hong Kong referred for reactions suggestive of β-lactam antibiotic allergy during the study period.

### Statistical Analysis

Data were extracted and analyzed using SPSS Statistics, version 20 (IBM Corp). Venn diagrams were created using jvenn.^27^ The χ^2^ statistic was used to calculate the association between clinical parameters and sensitization patterns among reagents in skin tests (ie, PPL, MD, benzylpenicillin, amoxicillin, and β-lactam antibiotics). A 2-sided *P* < .05 was considered statistically significant. The cumulative incidence and near-absolute prevalence were calculated by the number of patients divided by the total number of total estimated population of Hong Kong from January 1, 2018, and December 31, 2019. Population statistics from the Census and Statistics Department of the Hong Kong government was retrieved to estimate the coverage of the prevalence data.^28^

## Results

Complete records of a total of 7 184 271 unique patients were analyzed, representing more than 95% of the total estimated population of Hong Kong (7 524 100 in 2019), with a men to women ratio of 1:1.2 and with a median age of 44 years.^28^ The prevalence of β-lactam antibiotic allergy was 2.0% (n = 143 483) in more than 7.1 million patients.

**Figure 1.**
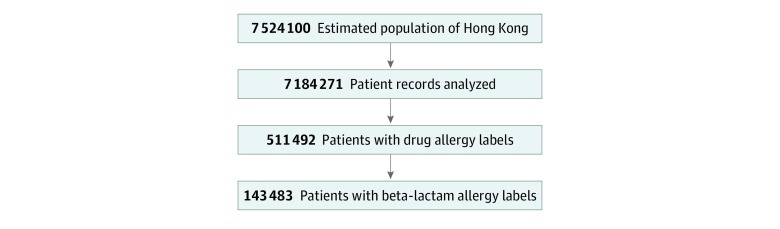
Prevalence of Reported Drug and β-lactam Antibiotic Allergies in Hong Kong

Of the 7 184 271 patients, 511 492 (7.1%) had physician-reported drug allergies, of which 143 483 (28.1%) were β-lactam antibiotic allergies. The point prevalence of β-lactam antibiotic allergy labels was therefore 2.0% (95% CI, 1.99%-2.01%), which is shown in Figure 1. The cumulative incidence was 107 per 100 000 population, with 8032 new β-lactam antibiotic allergies reported in 2018.

**Figure 2.**
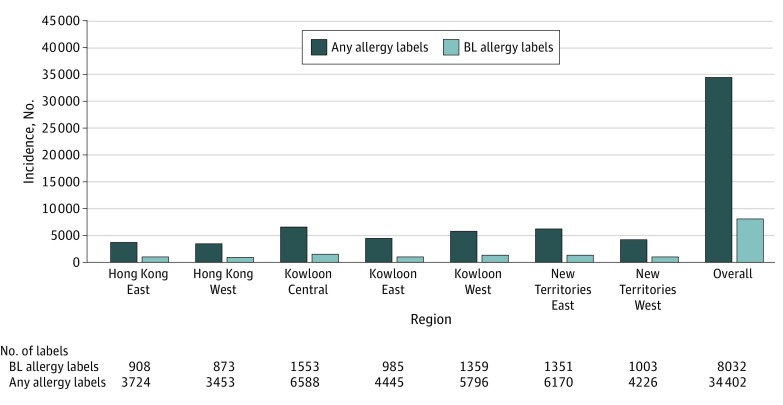
Incidence of New β-lactam Antibiotic Allergies in Different Regions in Hong Kong

A total of 34 402 new drug allergies were reported between January 1, 2018, and December 31, 2018. Figure 2 shows the incidence of new drug and β-lactam antibiotic allergies from each of the 7 clusters. Of the 34 402 new drug allergies reported in 2018, 8032 (23.3%) were β-lactam antibiotic allergies. The estimated cumulative incidence was therefore 107 (95% CI, 105.0-109.7) per 100 000 population during 2018 (based on the total estimated population of 7 486 400 in that year^28^).

Only 13.8% of all patients (n = 49) with reactions suggestive of a β-lactam antibiotic allergy had positive skin test results. More than 50% of patients (n = 35) were sensitized to PPL only and/or MD only.

**Figure 3.**
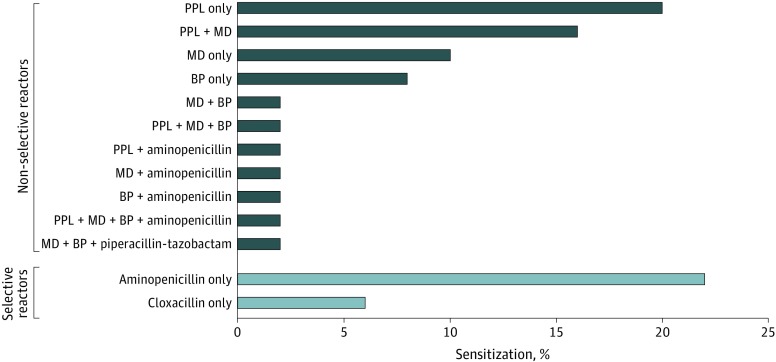
Sensitization Rates of Positive Skin Test Results BP indicates benzylpenicllin; MD, minor determinant; PPL, benzylpenicilloyl polylysine.

Skin tests were performed between 2018 to 2019 for 355 patients (part of the 7.1 million patient cohort) with reactions suggestive of β-lactam antibiotic allergies. Sensitization patterns of patients with positive skin test results are shown in Figure 3 and the Table.

**Table.** Skin Test Results of Patients With Reactions Suggestive of β-lactam Antibiotic Allergy

Skin Test Result	No./Total No. (%)
Negative result	306/355 (86.2)
Positive result	49/355 (13.8)
Selective reaction	14/49 (28.6)
Aminopenicillin	11/49 (22.4)
Cloxacillin sodium	3/49 (6.1)
Nonselective reaction	35/49 (71.4)
PPL or MD only	23/49 (47)
PPL only	10/49 (20.4)
MD only	5/49 (10.2)

Abbreviations: MD, minor determinant; PPL, benzylpenicilloyl polylysine.

The clinical parameters and skin test results of patients with positive results are shown in the eTable in the Supplement. No significant associations were found between the studied clinical parameters and sensitization patterns to PPL, MD, benzylpenicillin, aminopenicillin, and cloxacillin sodium.

The number of patients with positive skin test results was 49 (13.8%; 95% CI, 10.64%-17.90%). Of these patients, 14 (28.6%; 95% CI, 18.35%-44.49%) had a selective reaction and the remaining 35 (71.4%; 95% CI, 59.84%-85.27%) had a nonselective reaction. The sensitization rate to PPL or MD only was 47.0% (n = 23; 95% CI, 34.85%-63.21%), with 10 patients monosensitized to PPL only (20.4%; 95% CI, 11.74%-35.48%) and 5 to MD only (10.2%; 95% CI, 4.45%-23.42%).

## Discussion

To our knowledge, this cross-sectional study is the largest epidemiological study of reported β-lactam antibiotic allergies and the first to examine the sensitization pattern in patients in Hong Kong. The availability of the comprehensive Hospital Authority Clinical Management Systems made this study unique in that we were able to calculate the near-absolute prevalence and annual incidence of reported β-lactam antibiotic allergies for the entire territory. In addition, to our knowledge, this study is the first to report that patients in Hong Kong with β-lactam antibiotic allergy have much higher rates of sensitization to PPL and MD compared with western cohorts, highlighting the importance of incorporating these reagents in skin tests.

From more than 7.1 million records, the point prevalence of physician-reported β-lactam antibiotic allergies in Hong Kong was 2.0%. This percentage was much lower than the prevalence estimate of 5% reported in a pilot study cohort of hospitalized patients and was much lower than the rates in other western populations.^2,3,4,5,8^ This discrepancy may be attributed to the inherent differences between inpatients and the general population as well as between ethnicities and regions and to the lack of sampling bias. First, the incidence of antimicrobial allergies in hospitalized patients is known to be generally higher than the population means, with incidence of reported penicillin allergy reaching up to 15% in 1 report.^29^ This discrepancy likely reflects the greater use of β-lactam antibiotics in hospitalized patients, which further exacerbates patients’ risk of infection from multi–drug-resistant organisms and suboptimal therapy. Second, the present study was based on data of more than 95% of the entire population of Hong Kong, with patients who could seek care from 43 hospitals and 122 outpatient clinics, whereas the pilot report was based solely on a cohort of patients admitted to the acute general medical wards of a single hospital.^8^ The present study also had little sampling bias given that it included almost all patients (>95%) in Hong Kong, regardless of demographics or geography. Furthermore, all allergy data (either confirmed as no known drug allergy or as any other reaction suggestive of allergies) in the Clinical Management Systems can be entered by only the attending physicians after patient consultation. Patients often mistakenly self-report many non–immune-mediated adverse drug reactions as allergies.^9,10^ Therefore, because we used only the physician-reported allergy (an allergy can be recorded only after physician review of medical histories deemed it compatible with a genuine allergy), the data we collected likely have greater diagnostic accuracy compared with patient-reported allergies, which were often used in many studies of β-lactam antibiotic allergy.

The present study illustrates the power of comprehensive and accurate physician-reported big data and supersedes the pilot study.^8^ Future studies that use our approach or similar approaches may generate more accurate estimates of the true prevalence in different populations.

In addition to identifying the prevalence of β-lactam antibiotic allergies, this study delineated the incidence of new allergies reported per year. We found that, in 2018 alone, more than 26 000 new drug allergies and 8032 β-lactam antibiotic allergies were reported. These numbers translated to a cumulative incidence of more than 100 new β-lactam antibiotic allergies reported per 100 000 population each year. The incidence of new drug allergies is seldom studied and is likely an underappreciated phenomenon. Most self-reports of β-lactam antibiotic allergies are known to be false, and the pilot study found that after testing only approximately 10% of such reports were true allergies.^8^ If the rate of misreported allergies were true, more than 7200 patients may need an evaluation for potentially misreported new β-lactam antibiotic allergies in 2018 alone.

Robust and comprehensive epidemiological data, such as those used in the present study, are crucial to establishing allergy testing services and manpower requirements. For example, given that only a single public hospital in Hong Kong (Queen Mary Hospital) provides formal testing for any drug allergy and β-lactam antibiotic allergy, our data highlighted the urgent need to expand allergist training and allergy testing services throughout the territory.^30,31^ Region- and ethnicity-specific data are also essential to guide public awareness and health care professional (eg, paramedics, nurses, general practitioners, and specialists) education. Tailored interventions, such as nurse-led consultations and protocol-driven pathways, to expedite allergy testing are being implemented locally, and prospective studies are in progress. In addition, other centers should analyze the underreported incidence (rather than solely the prevalence) of new drug allergies in other populations.

Sensitization patterns among patients with β-lactam antibiotic allergy vary across different regions and ethnicities, which may be attributed to different prescribing practices and possible genetic or geographic predispositions. Regional variations in sensitization patterns have been well reported in non-Chinese populations, and substantial variability across different cohorts was found.^8^ This variability has substantial implications for skin testing methods and allergy practices. For example, a previous study involving a predominantly white cohort in the United Kingdom suggested that skin tests with PPL and MD could potentially be omitted in such populations with low sensitization rates.^18^ In this large cohort of patients with β-lactam antibiotic allergy in the UK, merely 8% of patients (8 of 99) were sensitized to PPL only and MD only.^18^ In countries where the complete panel of penicillin allergenic determinants (PPL, MD, benzylpenicillin, and amoxicillin) were commercially available and routinely incorporated in testing, similar rates of sensitization to PPL only and MD only were also reported; that is, 6% of patients (3 of 48) in Italy and 12% of patients (6 of 51) in Spain.^32,33^ The similarity in sensitization rate was attributed to higher use of amoxicillin in European countries.^18^ In the US, a decreasing rate of positive results from self-prepared penicillin skin test reagents was reported.^20^ Inversely, the present study found that 20.4% of patients were sensitized to PPL only and 10.2% of patients to MD only. Whether this finding was regionally or ethnically specific remains unclear. In the interim, we suggest that PPL and MD are essential components in β-lactam antibiotic allergy testing in Chinese populations. Especially because of the overwhelming need for β-lactam antibiotic allergy testing services, further studies into the optimal skin test strategies in specific populations are warranted.

### Limitations

This study has limitations that stem from its observational nature. For example, despite the large sample size, we were unable to retrieve additional demographic or clinical data from the Clinical Management Systems for further subgroup analysis because of data restrictions. Data other than for 2018 to 2019 were not available. We plan to perform further studies on long-term incidence rates in the future. For patients who underwent skin tests, potential selection bias may exist that could affect the generalizability of the findings. For example, clinicians may tend to refer patients with more severe reactions or to not refer patients with clear histories of genuine β-lactam antibiotic allergies. We tested only those patients who were referred for allergy investigation (regardless of any need for β-lactam antibiotic allergy testing) rather than systemically testing all patients with reported β-lactam antibiotic allergy. Furthermore, not all patients with negative skin test results had undergone drug provocation tests; therefore, we could not exclude the possibility of false-negative skin test results or definitely exclude allergy in patients with negative skin test results. Although the number of patients with positive skin test results was relatively small, overestimating or underestimating the sensitization rates was possible, highlighting the necessity of future prospective studies.

## Conclusions

To our knowledge, this study is the largest epidemiological analysis of reported β-lactam antibiotic allergies. In Hong Kong, the near-absolute prevalence of physician-reported β-lactam antibiotic allergies was 2.0% of the 7.1 million patient records we examined. The cumulative incidence was 107 per 100 000 population, with more than 8000 new β-lactam antibiotic allergies reported in 2018 alone. Unlike Europeans, Chinese patients in Hong Kong with β-lactam antibiotic allergy had much higher monosensitization rates to PPL and MD, making these reagents essential in β-lactam antibiotic skin tests. Future studies are necessary to ascertain whether this phenomenon remains ethnicity or region specific.

## Notes

Supplement. **eTable.** Clinical Characteristics and Skin Test Results of 49 Patients With Positive Skin Tests
