# Supplementary material for: Prevalence, Incidence, and Sensitization Profile of β-lactam Antibiotic Allergy in Hong Kong
Source: JAMA Netw Open. 2020 May 6;3(5):e204199. doi: 10.1001/jamanetworkopen.2020.4199 (PMC7203606; doi:10.1001/jamanetworkopen.2020.4199)
Supplement: Supplement. — eTable. Clinical Characteristics and Skin Test Results of 49 Patients With Positive Skin Tests [file jamanetwopen-3-e204199-s001.pdf]

Supplementary Online Content

Li PH, Yeung HHF, Lau C-S, Au EYL. Prevalence, incidence, and sensitization profile of β-lactam antibiotic allergy in Hong Kong. *JAMA Netw Open.* 2020;3(5):e204199. doi:10.1001/jamanetworkopen.2020.4199

eTable. Clinical Characteristics and Skin Test Results of 49 Patients With Positive Skin Tests

This supplementary material has been provided by the authors to give readers additional information about their work.

©2020 Li PH et al. *JAMA Network Open*

eTable. Clinical Characteristics and Skin Test Results of 49 Patients With Positive Skin Tests

Patient	Interval between reaction and testing (years)	Index beta-lactam	Type of reaction	Skin test results				
				PPL	MD	BP	AMOX	Other (specified if +)
1	5	Amoxicillin-clavulanate	Immediate	+	-	-	-	
2	8	Amoxicillin-clavulanate	Immediate	+	-	-	+	
3	>10	Amoxicillin	Delayed	+	-	-	-	
4	>10	Unknown	Unknown	+	-	-	-	
5	>10	Unknown	Delayed	+	-	-	-	
6	2	Amoxicillin-clavulanate	Delayed	-	-	-	+	
7	>10	Unknown	Unknown	+	+	-	-	
8	7	Amoxicillin-clavulanate	Delayed	+	-	-	-	
9	2	Unknown	Delayed	-	+	-	-	
10	1	Unknown	Immediate	-	+	-	-	
11	4	Ampicillin	Immediate	+	+	-	-	
12	<1	Amoxicillin-clavulanate	Immediate	+	+	-	-	
13	<1	Amoxicillin-clavulanate	Immediate	-	+	+	-	
14	6	Unknown	Delayed	-	-	-	+	
15	>10	Benzylpenicillin	Delayed	-	+	-	-	
16	>10	Cloxacillin	Delayed	-	-	-	-	Cloxacillin
17	5	Amoxicillin-clavulanate	Immediate	+	-	-	-	
18	<1	Cefazolin	Immediate	+	+	-	-	
19	2	Benzylpenicillin	Immediate	-	-	+	-	
20	2	Amoxicillin-clavulanate	Delayed	-	-	-	+	
21	<1	Amoxicillin-clavulanate	Immediate	-	+	+	-	

©2020 Li PH et al. *JAMA Network Open*

22	5	Ampicillin & Cloxacillin	Immediate	+	+	-	-	
23	<1	Amoxicillin-clavulanate	Immediate	+	-	-	-	
24	<1	Unknown	Immediate	-	+	-	-	
25	1	Cefazolin	Immediate	+	+	-	-	
26	10	Cefalexin	Immediate	-	+	-	-	
27	<1	Amoxicillin-clavulanate	Immediate	-	-	-	+	
28	>10	Unknown	Unknown	+	-	-	-	
29	<1	Amoxicillin-clavulanate	Immediate	-	-	+	+	
30	<1	Cloxacillin	Immediate	-	-	-	-	Cloxacillin
31	<1	Amoxicillin-clavulanate	Immediate	+	+	+	-	
32	<1	Amoxicillin-clavulanate	Immediate	-	-	-	+	
33	2	Unknown	Unknown	+	-	-	-	
34	>10	Ampicillin & Cloxacillin	Unknown	+	+	-	-	
35	<1	Amoxicillin-clavulanate	Immediate	-	-	+	-	
36	<1	Amoxicillin-clavulanate	Immediate	+	+	+	+	
37	4	Ampicillin & Cloxacillin	Immediate	-	-	-	-	Ampicillin
38	1	Amoxicillin	Immediate	-	+	-	+	
39	>10	Amoxicillin-clavulanate	Delayed	+	+	-	-	
40	1	Ampicillin	Immediate	-	-	-	+	
41	1	Piperacillin-tazobactam	Immediate	-	+	+	-	Piperacillin-tazobactam
42	>10	Cloxacillin	Unknown	-	-	-	-	Cloxacillin
43	2	Unknown	Unknown	+	-	-	-	
44	3	Amoxicillin-clavulanate	Immediate	-	-	+	-	
45	2	Amoxicillin-clavulanate	Immediate	-	-	-	+	
46	>10	Benzylpenicillin	Delayed	-	-	+	-	
47	>10	Unknown	Delayed	-	-	-	+	
48	5	Amoxicillin-clavulanate	Immediate	-	-	-	+	
49	<1	Ampicillin	Delayed	-	-	-	-	Ampicillin

©2020 Li PH et al. *JAMA Network Open*

PPL: benzylpenicilloyl-poly-l-lysine, MD: minor determinant, BP: benzylpenicillin, AMOX: amoxicillin,

+: Positive, -: Negative

©2020 Li PH et al. *JAMA Network Open*
